# A CROSS-COUNTRY ANALYSIS OF COGNITION AND ASSOCIATED RISK FACTORS

**DOI:** 10.1093/geroni/igad104.0947

**Published:** 2023-12-21

**Authors:** David Knapp, Arie Kapteyn, Alessandro Giambrone, Tabasa Ozawa

**Affiliations:** University of Southern California, Los Angeles, California, United States; University of Southern California, Los Angeles, California, United States; University of Southern California, Los Angeles, California, United States; University of Southern California, Los Angeles, California, United States

## Abstract

The widely cited Lancet Commission (Livingston et al., 2017) concluded that a third of dementia cases may be preventable through appropriate interventions targeting what they refer to as modifiable risk factors. These risk factors have been widely studied individually, but rarely investigated collectively and across many countries. We analyze the cross-country consistency of relationships between these modifiable risk factors and cognition using an internationally comparable set of aging studies in 31 countries including the United States, England and Europe. Cross-country differences in culture, policies, economy, and other collective experiences lead to significant variation in lifecycle outcomes, including dementia onset and modifiable risk factors. We find a limited number of robust relations: education, depression, and hearing show clear, consistent associations with our cognition measure, the sum of immediate and delayed recall. The evidence for other factors, including obesity, smoking, diabetes, and hypertension is weaker and becomes almost non-existent when correcting for multiple hypotheses testing. The inconsistent relationship across countries between these risk factors and cognition suggests the lack of a causal mechanism leading to cognitive decline – a necessary condition for these risk factors to be modifiable and effective targets for policy interventions aimed at controlling the prevalence and cost of dementia.

